# Cell morphology-based machine learning models for human cell state classification

**DOI:** 10.1038/s41540-021-00180-y

**Published:** 2021-05-26

**Authors:** Yi Li, Chance M. Nowak, Uyen Pham, Khai Nguyen, Leonidas Bleris

**Affiliations:** 1 Bioengineering Department, University of Texas at Dallas, Richardson, TX USA; 2 Center for Systems Biology, University of Texas at Dallas, Richardson, TX USA; 3 Department of Biological Sciences, University of Texas at Dallas, Richardson, TX USA

**Keywords:** Biomedical engineering, Computer modelling

## Abstract

Herein, we implement and access machine learning architectures to ascertain models that differentiate healthy from apoptotic cells using exclusively forward (FSC) and side (SSC) scatter flow cytometry information. To generate training data, colorectal cancer HCT116 cells were subjected to miR-34a treatment and then classified using a conventional Annexin V/propidium iodide (PI)-staining assay. The apoptotic cells were defined as Annexin V-positive cells, which include early and late apoptotic cells, necrotic cells, as well as other dying or dead cells. In addition to fluorescent signal, we collected cell size and granularity information from the FSC and SSC parameters. Both parameters are subdivided into area, height, and width, thus providing a total of six numerical features that informed and trained our models. A collection of logistical regression, random forest, k-nearest neighbor, multilayer perceptron, and support vector machine was trained and tested for classification performance in predicting cell states using only the six aforementioned numerical features. Out of 1046 candidate models, a multilayer perceptron was chosen with 0.91 live precision, 0.93 live recall, 0.92 live *f* value and 0.97 live area under the ROC curve when applied on standardized data. We discuss and highlight differences in classifier performance and compare the results to the standard practice of forward and side scatter gating, typically performed to select cells based on size and/or complexity. We demonstrate that our model, a ready-to-use module for any flow cytometry-based analysis, can provide automated, reliable, and stain-free classification of healthy and apoptotic cells using exclusively size and granularity information.

## Introduction

Since its invention in 1960s, fluorescence-based flow cytometry has become one of the most powerful tools in biomedical research to efficiently and quantitatively analyze information of cellular properties at single-cell level, which range from cell counting and sorting to determining cell characteristics and states^1–3^. Typically, the processing of the samples commences by selecting cells based on their size and granularity, with a general gating strategy based on the forward scatter (FSC) and side scatter (SSC) values, which are correlated with the size and granularity of the cells, respectively^4,5^. This process assists in separating healthy cells from apoptotic ones based on morphological changes, which for example may result from adverse conditions during cell suspension preparations. Notably, FSC and SSC yield three related measurements (A: area, H: height, W: width). In common practice, only one pair of the readings (e.g., FSC-A vs. SSC-H) is used to manually determine a gating boundary.

Identifying the state of cells (i.e., healthy vs. apoptotic) can be implemented with high accuracy using a conjugated Annexin V/propidium iodide (PI)-staining assay. Specifically, Annexin V belongs to a family of calcium-dependent phospholipid-binding proteins, which has a strong binding affinity for phosphatidylserine (PS). Under normal physiological conditions, PS is internalized to the inner leaflet of the cell membrane. Upon apoptosis initiation, PS is translocated to the outer leaflet of the cell membrane in an ATP-dependent manner to become accessible for signal detection by phagocytic cells^6^. Thus, by conjugating a fluorophore to Annexin V, early apoptotic cells can be detected with flow cytometry^7^. Furthermore, as cells continue to progress through apoptosis the cell membrane begins to lose integrity and cells become necrotic. The fluorescent viability dye, PI, is usually impermeable to the cell membrane, but as the cell begins to degenerate, PI freely diffuses into and stains nucleic acids within the dying cell; thus, distinguishing early and late apoptotic/necrotic cells.

Machine learning refers to the construction of statistical models based on sample data (training data) in order to make predictions or decisions on future data (testing data). Due to the rapidly developing computational infrastructure, applications for machine learning in the past two decades have witnessed an explosive growth in diverse knowledge domains including biomedical research and medicine^8–10^. Prominent examples include cancer prognosis/prediction^11–13^, drug discovery and development^14–16^, as well as deciphering complex biological networks^17–19^.

Since 2007, several machine learning-based computational approaches have been proposed to differentiate cell types and states using microscopy^20–33^. As an example, using time-lapse data of cell mass distribution acquired from the quantitative phase imaging (QPI) assay, Balvan and colleagues devised a Bidirectional Long Short-term Memory network that achieved 76% accuracy for cell death detection^34^. Similarly, based on the morphological features extracted from the fluorescence microscopy images, Duever and colleagues built a support vector machine (SVM) classifier to distinguish between normal and apoptotic states of CHO cells^35^.

Each of the above experimental and computational protocols to study cell health has its own disadvantages. The Annexing V/PI staining method, despite being highly accurate, is relatively laborious and expensive. Importantly, the staining process may interfere with measurements of other cellular parameters. Unfortunately, the comparatively straightforward FSC-A/SSC-H gating protocol involves arbitrary boundary drawing and thus may introduce human bias. Furthermore, the current computational methods for live/apoptotic cell prediction mostly rely on phase contrast or fluorescence microscopy images, which may not be a viable option in many studies.

To address these challenges, we hypothesize that each of the six FSC and SSC measurements (FSC-A, FSC-H, FSC-W, SSC-A, SSC-H, and SSC-W) contains unique information about the cellular state, and presumably their combination can be indicative of apoptotic state. Accordingly, using these six-cell morphology-based features, we have successfully built a Multilayer Perceptron (MLP)-based predictive model which returns both high precision and high recall when predicting the live cells within a population.

## Results

### Data collection for training and testing sets

The human colorectal cancer cell line HCT116 was reverse transfected with miR-34a-5p mimic (final concentration: 25 nM)^36^. The cells were then stained with Annexin V-Alexa Fluor 488 dye and PI before being analyzed by flow cytometry^37^. Live and apoptotic cells were distinguished with a cutoff value from Annexin V-Alexa Fluor 488 signal (Supplementary Figs. 1 and 2, i.e., Alexa Fluor 488-negative cells were considered live, while Alexa Fluor 488-positive were considered apoptotic). A small percentage of the cells was found to contain negative values for Alexa Fluor 488 and PI fluorescence and were subsequently excluded from our analysis. In total, 9990 cells were recovered, which contained 5722 live cells and 4268 apoptotic cells. To ensure the balance between the two labels (the ratio of live and apoptotic cells = 1), we then randomly sampled 4268 live cells from the original live cell population, which were combined with the 4268 apoptotic cells to form the starting dataset. Finally, this starting dataset was randomly split into the training dataset and testing dataset at a ratio of 80:20 (size of training dataset: size of testing dataset). Specifically, the training dataset (6828 cells, Supplementary Table 1) contained 3411 live cells and 3417 apoptotic cells. The testing dataset (1708 cells, Supplementary Table 2) contained 857 live cells and 857 apoptotic cells.

For the purposes of classification for machine learning training sets, the “live” state was labeled as 0, while the “apoptotic” state as 1. In addition to the signal collected for the apoptotic dyes, FSC-A, FSC-H, FSC-W, SSC-A, SSC-H, and SSC-W values were also collected and served as the features for building the predictive models.

### Data preprocessing and visualization

**Fig. 1:**
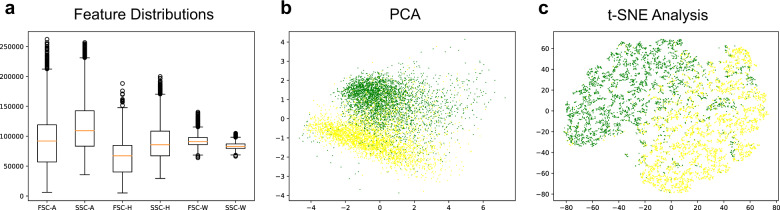
Descriptive statistics and visualization of the training data. **a** Box and whisker plot of the training dataset. The means of the six features (FSC-A, SSC-A, FSC-H, SSC-H, FSC-W, and SSC-W) were comparable, with the maximal ratio less than 2.0-fold (mean_SSC-A_/mean_FSC-H_ = 1.81). **b** Visualization of six-dimensional training dataset (green: live cells, yellow: apoptotic cells) using PCA (principal component analysis). **c** Visualization of the six-dimensional training dataset (green: live cells, yellow: apoptotic cells) using t-SNE (t-distributed stochastic neighbor embedding).

We first explored the statistical distributions of the six features (FSC-A, SSC-A, FSC-H, SSC-H, FSC-W, and SSC-W) using box plotting. As shown in Fig. 1a and Supplementary Table 3, the means of these features were comparable, with the maximal ratio less than 2.0-fold (mean_SSC-A_/mean_FSC-H_ = 1.81). Nevertheless, since we adopted Euclidean distance-based learning algorithms (e.g., k-NN: k-nearest neighbors), and the scaling operations were known to speed up gradient descent and thus facilitate algorithms, such as MLP, we subjected our training and testing samples to data standardization and compared the performances of models derived from both non-standardized and standardized datasets.

In order to visualize the training samples in two-dimensions, we applied both principal component analysis (PCA) and t-distributed stochastic neighbor embedding t-SNE^38^ to the standardized training data using two components. As shown in Fig. 1b (PCA) and 1c (t-SNE), the live (green) and apoptotic (yellow) cells were clearly separable, yet significant overlapping still occurred centered at (0, 0) positions.

### Selection of predictive models

**Fig. 2:**
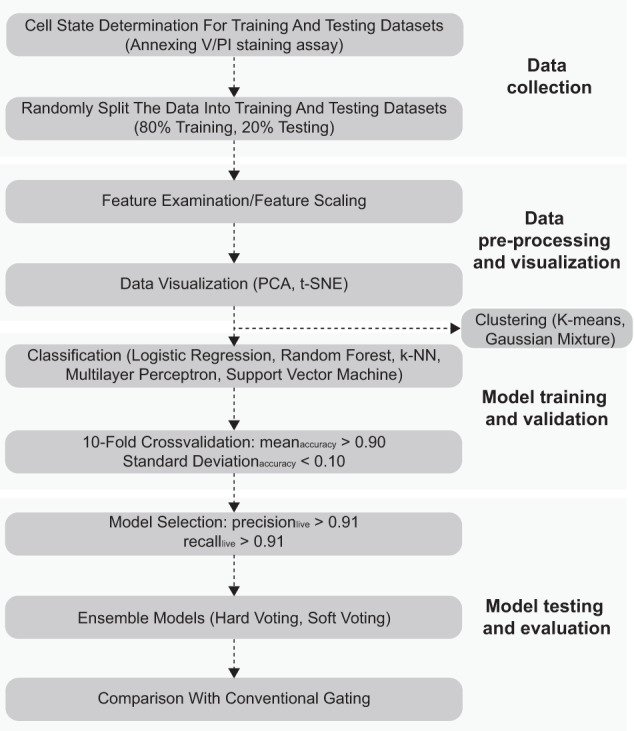
General workflow of building a cell morphology-based machine learning model. The modeling process consists of four steps: Data collection, Data pre-processing and visualization, Model training and validation, and Model testing and evaluation.

Considering our number of features as well as the uncertainty of linearity or non-linearity, we subject the training dataset to five classification algorithms (logistical regression, random forest, k-nearest neighbor, MLP, and SVM) in order to systematically compare their predictive performances (for general workflow, see Fig. 2). The Naïve Bayes classifier was not included because some features, such as FSC-W and FSC-H, are known to be biologically related. Specifically, all screening models were subjected to tenfold cross-validation based on two filtering criterions to ensure acceptable balance between model bias and variance: (1) the mean accuracy >0.90, and (2) the standard deviation of accuracy <0.10. Using standardized training data, the logistical regression model yielded a mean accuracy value less than 0.90 and was discarded. In contrast, 93 random forest screening models (number of trees from 1 to 100, Supplementary Table 4) satisfied these two filtering conditions. Similarly, 79 k-NN screening models (*k* = 1–100, Supplementary Table 5) were preserved. For MLP, we fixed the number of hidden layers of the neural network at 2, as it has been proposed that a 2-hidden layer model could approximate any arbitrary smooth mapping to any given accuracy. Out of 900 screening MLP models (number of nodes for the first hidden layer = 1–30, number of nodes for the second hidden layer = 1–30), 862 passed the given filter and were subjected to further analysis (Supplementary Table 6). For SVM models, three kernels (Linear, Gaussian, and Sigmoid) functions and a range of their corresponding parameters (C: cost parameter, gamma for Gaussian kernel) were included (details in “Materials and Methods/Machine learning model training and testing” sections), and for the standardized training set, 12 models using the Gaussian kernel passed the given filter (Supplementary Table 7).

We subsequently applied the 1046 candidate models (93 for random forest, 79 for k-NN, 862 for MLP, and 12 for SVM) to the standardized testing data and further filtered the models based on: (1) precision for predicting the live cell population >0.91, and (2) recall for predicting the live cell population >0.91. Our focus is mainly on the predictive power of the live population, as this study was primarily designed for improving “gating” of live cells in general flow cytometry-based assays without the need for staining. As shown in Supplementary Table 8, only 96 MLP models survived this additional filter, and 3 of those models with AUC values >0.97 (number of nodes for first layer = 13/number of nodes for second layer = 19, number of nodes for first layer = 13/number of nodes for second layer = 21, and number of nodes for first layer = 16/number of nodes for second layer = 6, named as MLP 13-19, MLP 13-21, and MLP 16-6, respectively) were included in our final candidate classification models (Table 1).

**Table 1:** Predictive performances of the three candidate learning models.

Index	True live	False apoptotic	False live	True apoptotic	Live precision	Live recall	Live *f* value	Accuracy	ROC_AUC	Average precision	Tenfold cross-validation accuracy standard deviation	Tenfold cross-validation accuracy mean
MLP 13-19	798	59	76	775	0.913	0.931	0.922	0.921	0.970	0.972	0.006	0.918
MLP 13-21	795	62	72	779	0.917	0.928	0.922	0.922	0.970	0.972	0.007	0.917
MLP 16-6	800	57	77	774	0.912	0.933	0.923	0.922	0.970	0.973	0.008	0.917

In parallel, we performed the identical analytic procedures on non-standardized training data. The logistical regression, all of 100 k-NN (*k* = 1–100), and all of 68 SVM models failed to yield mean accuracy larger than 0.90 and standard deviation of accuracy smaller than 0.10 during the tenfold cross-validation analysis. In contrast, 94 random forest (number of trees from 1 to 100) and one MLP model satisfied this initial filtering condition. However, when subsequently applied to the testing dataset, all of these models (95 in total) failed to pass the second filtering condition (precision > 0.91 and recall > 0.91 when predicting live cells). As an example, although the model MLP 19-4 (number of nodes in the first layer: 19, number of nodes in the second layer: 4) passed the first filtering, it yielded relatively low precision when predicting live cells on the testing dataset (precision: 88.8%). These results indicate that the magnitudes of features in our dataset varied significantly, which makes feature scaling (e.g., normalization and standardization) essential for optimizing the data training process.

### Comparison of candidate models

**Fig. 3:**
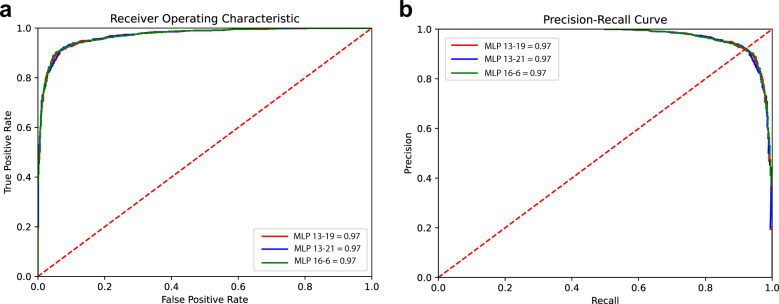
Comparison of predictive performances of the three candidate models. **a** ROC curves of the candidate models. **b** Precision-recall curves of the candidate models. Both ROC and Precision-recall analysis indicated that the three multilayer perceptron-based models yielded comparable performance when predicting live cells.

To compare the three candidate models, we plotted both the receiver operating characteristic (ROC) and precision-recall curves. As shown in Fig. 3a, the three MLP models (MLP 13-19, MLP 13-21, and MLP 16-6) displayed comparable AUC values (0.97) in the ROC plot. In addition, the three models showed identical average precision values (Fig. 3b, average precision: 0.97), indicating that all three models performed with similar strength when predicting the live cell subpopulation.

We further explored whether ensemble models based on the three candidate models could produce more accurate predictive outcomes. To this end, we subjected the models to hard voting, which makes the prediction based on a simple majority vote, and soft voting, which averages the probabilities derived from individual models. As shown in Table 2, when comparing the individual model with highest ROC AUC value (MLP 16-6), both hard voting and soft voting marginally improved the precision when predicting the live cells (91.7% for hard voting, 91.3% for soft voting, and 91.2% for MLP 16-6), while slightly decreasing recall values (93.2% for hard voting, 93.0% for soft voting, and 93.3% for MLP 16-6). Taken together, these observations indicated that the ensemble models did not significantly improve the precision (Supplementary Fig. 3) and recall values for the live cell population. This was possibly because all original models had already shown relatively high predictive power (the ROC AUC values for all three models were larger than 0.97).

**Table 2:** Predictive performances of the ensemble models based on the three candidate models using two labels (0: live, 1: apoptotic).

Index	True live	False apoptotic	False live	True apoptotic	Live precision	Live recall	Live *f* value	Accuracy	ROC_AUC
MLP 16-6	800	57	77	774	0.912	0.933	0.923	0.922	0.970
hard_vote	799	58	72	779	0.917	0.932	0.925	0.924	N/A
soft_vote	797	60	76	775	0.913	0.930	0.921	0.920	0.971

**Fig. 4:**
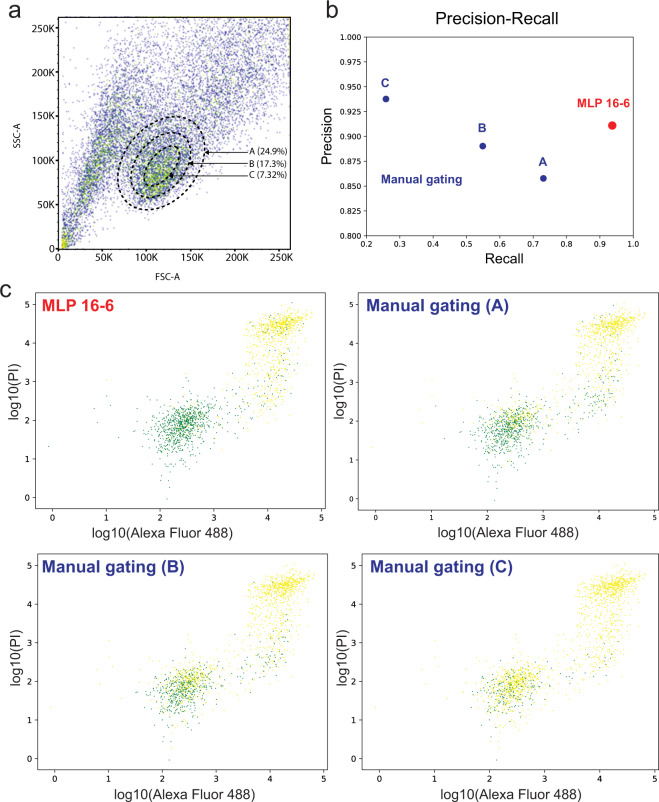
Comparison between the MLP 16-6-based and manual gating. **a** Manual gating of “live” cells using SSC-A and FSC-A. Three gates of different size were included and the percentage of “live” cells for each gate was calculated. **b** Comparison of precisions and recalls of live cell predictions between the MLP 16-6-based and conventional gating methods. **c** Classifications of live (green) and apoptotic (yellow) cells using manual gating and MLP 16-6-based gating. The x-axis denotes log_10_(Alexa Fluor 488), and the y-axis denotes log_10_(PI).

Finally, the FSC-A vs. SSC-A gating method is typically used to isolate healthy cells (three gates of different size, Fig. 4a) based on size and granularity (complexity). As shown in Fig. 4b, this conventional approach results in both lower precision (85.8% for gate A, 89.1% for gate B, compared to 91.2% for MLP 16-6) and much lower recall values (73.6% for gate A, 54.5% for gate B, compared to 93.3% for MLP 16-6) for gates A and B when predicting live cells. In addition, while the gate C (the smallest size) yielded a slightly higher precision (93.9%), it failed to recover the vast majority of true live cells (recall: 25.5%). As evident in Supplementary Figs. 1 and 2, the poor performance of the manual gating mainly arose from the significant overlapping and co-existence of live and apoptotic cells within the main cell cluster in a FSC-A vs. SSC-A plot (green: live, yellow: apoptotic). Conceivably, the exclusion of a relatively large percentage of live cells (Fig. 4c) after conventional gating, and to a lesser extent the inclusion of apoptotic cells, will affect the validity of subsequent cellular analysis^39,40^, especially in the context of cell health-related assays, such as cell proliferation or drug response.

## Discussion

There are two main types of machine learning approaches: supervised and unsupervised learning. Supervised learning requires using predefined labels (e.g., live or apoptotic cell state) that are assigned to experimental instances (training dataset). Whereas in unsupervised learning, no such labels are available and instead similar instances are grouped (clustered) based on the inherent structure of their features. To compare the performance between these two approaches, we further subjected our standardized or non-standardized training data to two commonly used clustering algorithms (K-means clustering and Gaussian mixture clustering, number of clusters = 2). As shown in Supplementary Figs. 4–7, both algorithms delivered relatively poor predictive performances on both standardized and non-standardized training data, as seen by the extensive overlap of the two clusters from both live and apoptotic cell populations. When using the best-performing labeling schema for non-standardized data, the K-mean clustering (green for live, and red for apoptotic cells) yielded 52.7% of precision, 41.3% of recall, and 52.2% of accuracy when predicting the live cells (Supplementary Fig. 4), and the Gaussian mixture clustering (green for apoptotic, and red for live cells) yielded 57.4% of precision, 65.1% of recall, and 58.5% of accuracy (Supplementary Fig. 5). Similarly, for standardized data, the K-mean clustering (green for live, and red for apoptotic cells) yielded 50.3% of precision, 62.8% of recall, and 50.4% of accuracy when predicting the live cells (Supplementary Fig. 6), and the Gaussian mixture clustering (green for apoptotic, and red for live cells) yielded 57.5% of precision, 65.1% of recall, and 58.5% of accuracy (Supplementary Fig. 7). These findings corroborate a general rule in machine learning stating that unless necessary, relevant data (e.g., the cell state labels) should not be discarded.

As described above, the Annexin V/PI assay is used to not only separate live and apoptotic cells, but additionally differentiate between early and late apoptotic cell subpopulations (i.e., live cells: Annexin V-negative and PI-negative, early apoptotic cells: Annexin V-positive and PI-negative, late apoptotic cells which include necrotic and dying/dead cells: Annexin V-positive and PI-positive). Thus, for the same miR-34a-treated HCT116 cells, we first removed any cells with negative values for Alexa Fluor 488 and PI fluorescence. In total, 9990 cells were recovered, which contained 5722 live cells (labeled as 0), 699 early apoptotic cells (labeled as 1), and 3569 late apoptotic cells (labeled as 2). Next, this starting dataset was randomly split into the training dataset and testing dataset at a ratio of 80:20 (size of training dataset: size of testing dataset). Specifically, the training dataset (7992 cells, Supplementary Table 9) contained 4607 live cells, 552 early apoptotic cells, and 2 833 apoptotic cells. The testing dataset (1998 cells, Supplementary Table 10) contained 1115 live cells, 147 early apoptotic cells, and 736 apoptotic cells (Supplementary Fig. 8). Both datasets were subsequently standardized as described above. To ensure the balance between the three labels (the ratio of live, early, and late apoptotic cells = 1) in the training dataset, we then used the SMOTE (Synthetic Minority Over-sampling Technique) algorithm to create synthetic samples for minority classes (early and late apoptotic cells). Consequently, the resulting training dataset contained the equal number of samples for all three labels (4607 samples for each label, Supplementary Table 11). Meanwhile, it should be noted that when constructing predictive models, oversampling and generation of synthetic observations shall be applied to the training dataset alone, and not to the testing dataset^41^.

Next, we constructed both random forest (number of trees from 1 to 100) and MLP (number of nodes for the first hidden layer = 1–30, number of nodes for the second hidden layer = 1–30) models using the balanced/standardized training dataset, and subsequently applied all these 1000 models on the testing dataset using the same filtering condition (precision > 0.91 and recall > 0.91 when predicting live cells). Interestingly, all surviving models (5 for MLP, Supplementary Table 12, and 49 for random forest, Supplementary Table 13) showed relatively good predictive performance for both live and late apoptotic cell subpopulations. As an example, for the MLP model with highest accuracy value (88.5%, MLP 7-2), it predicted the live cells with 93.2% precision and 91.1% recall. In addition, it yielded 91.5% precision and 89.7% recall when predicting late apoptotic cells. Similarly, for the random forest model with highest accuracy value (88.3%, RF 76), it predicted the live cells with 93.0% precision and 91.4% recall, and additionally predicted the late apoptotic cells with 91.4% precision and 89.1% recall. In contrast, both models demonstrated poor predictive power for early apoptotic cells (Supplementary Figs. 9 and 10, precision for MLP 7-2: 49.7%, precision for RF 76: 48.4%, recall for MLP 7-2: 63.3%, recall for RF 76: 60.5%), and it should be noted that the ensemble models (hard voting or soft voting) based on these two models did not yield better predictive outcomes for the early apoptotic cells. Specifically, both hard and soft voting marginally increased the precision when predicting the early apoptotic cells (hard voting: 50.9%, soft voting: 51.7%), while slightly decreasing the recall values (hard voting: 59.2%, soft voting: 61.2%) compared to the MLP 7-2 model (Table 3). This deficiency may arise from the fact that compared to live and late apoptotic cells, the original training dataset contained much less early apoptotic cells. In addition, it is conceivable that as a transitional stage, the early apoptotic cells may inherently resemble either live or late apoptotic cells morphologically.

**Table 3:** Predictive performances of the ensemble models based on the two candidate models using three labels (0: live, 1: early apoptotic, 2: late apoptotic).

Candidate Model	m00	m01	m02	m10	m11	m12	m20	m21	m22	Live precision	Live recall	Live *f* value	Early precision	Early recall	Early *f* value	Late precision	Late recall	Late *f* value	Accuracy
RF 76	1019	57	39	35	89	23	42	38	656	0.930	0.914	0.922	0.484	0.605	0.538	0.914	0.891	0.902	0.883
MLP 7-2	1016	62	37	30	93	24	44	32	660	0.932	0.911	0.922	0.497	0.633	0.557	0.915	0.897	0.906	0.885
hard_vote	1056	35	24	39	87	21	55	49	632	0.918	0.947	0.932	0.509	0.592	0.547	0.934	0.859	0.895	0.888
soft_vote	1026	50	39	34	90	23	42	34	660	0.931	0.920	0.926	0.517	0.612	0.561	0.914	0.897	0.905	0.889

As mentioned earlier, the field of cytology has witnessed great advances in the past decade in terms of both single-cell instrumentation and machine learning-based data analysis^20–35^. As an example, Nitta et al. established an intelligent image-activated cell sorter which integrates high-throughput cell microscopy, focusing, and sorting on a hybrid software-hardware data-management infrastructure^31^. Using this platform, they were able to separate platelet aggregates from human blood sample with extremely high precision (99.0%). Similarly, Lee et al.^23^ devised a multiplexed asymmetric-detection time-stretch optical microscopy (multi-ATOM) that can accommodate ultrahigh-throughput (>700,000 cells/s) single-cell biophysical phenotyping at high accuracy (>94%). Although most of these studies focused on differentiating cell types, it is conceivable that similar approaches could be adopted to determine cell heath states.

Compared to our protocol, which is based on standard flow cytometry, these technologies capture more information of physically measurable quantities (features) of the cells and thus could potentially allow more thorough and accurate cell profiling. As an example, using an intelligent frequency-shifted optofluidic time-stretch quantitative phase imaging (OTS-QPI) platform^30^, Wu et al. successfully separated white blood cells from HL-60 leukemia cells with both 99% precision and 99% recall. Nevertheless, we emphasize that many of these platforms are highly customized and complex (e.g., the OTS-QPI^30^ system consists of both a frequency-shifted OTS-QPI microscopy and a microfluidic chip, Supplementary Table 14), and thus may not be readily available to research labs compared to conventional flow cytometry. As a consequence, we argue that our methodology offers an accurate and easy-to-use live cell identification approach using a simple flow cytometry-based assay. In addition, our model (MLP 16-6) provides comparable or even higher predictive performance compared to several image-based predictive algorithms. As an example, the SVM model proposed by Duever and colleagues^35^ yielded 86.5% of precision and 90.0% of recall when predicting live cells, both of which were significantly lower than our MLP 16-6 model (Supplementary Table 14).

In conclusion, we developed a MLP-based machine learning model (MLP 16-6) based on six FSC and SSC-related cell properties that provides higher predictive performance compared to the conventional FSC-A/SSC-A gating method and is relatively inexpensive compared to the Annexin V/PI apoptotic classification assay. We envision our algorithm will provide a convenient and accurate alternative for various flow cytometry-based biological assays.

## Materials and methods

### Mammalian cell culture

The HCT116 cells were acquired from the American Type Culture Collection (catalog number: CCL-247) and maintained at 37 °C, 100% humidity and 5% CO_2_. The cells were grown in Dulbecco’s modified Eagle’s medium (Invitrogen, catalog number: 11965–1181) supplemented with 10% fetal bovine serum (Invitrogen, catalog number: 26140), 0.1 mM MEM non-essential amino acids (Invitrogen, catalog number: 11140–050), and 0.045 units/mL of Penicillin and 0.045 units/mL of Streptomycin (Penicillin-Streptomycin liquid, Invitrogen, catalog number: 15140). To pass the cells, the adherent culture was first washed with PBS (Dulbecco’s Phosphate Buffered Saline, Mediatech, catalog number: 21-030-CM), then trypsinized with Trypsin-EDTA (0.25% Trypsin with EDTAX4Na, Invitrogen, catalog number: 25200) and finally diluted in fresh medium.

### Apoptosis induction and staining

To generate the miR-34a-treated testing dataset, HCT116 cells were reverse transfected with miR-34a-5p mimic (Qiagen, catalog number: MSY0000255, final concentration: 25 nM) with Lipofectamine RNAiMAX (ThermoFisher catalog number: 13778075) and allowed to incubate for 48 h. Subsequently, cells were detached from 100 mm Petri Dish by aspirating off the growth medium, washing with 10 mL of PBS (Mediatech, catalog number: 21-030-CM), trypsinizing the attached cells with 2 mL 0.25% Trypsin-EDTA (Invitrogen, catalog number: 25200), then quenched with fresh complete media. Cells were centrifuged at 1000 rpm for 5 min at room temperature. The cell pellets were then washed with 1 mL of PBS, again centrifuged at 1000 rpm for 5 min at room temperature before finally being resuspended in 1X annexin-binding buffer. Next, the resuspended cells were stained using the Dead Cell Apoptosis Kit with Annexin V-Alexa Fluor™ 488 & PI (Invitrogen, catalog # V13241), following the manufacturer’s instructions. The samples were then analyzed on a LSR Fortessa (BD Biosciences) flow cytometer. Excitation/emission wavelengths for the annexin V-Alexa Fluor™ 488 conjugate is 495/519 nm, and 533/617 nm for PI.

### Machine learning model training and testing

Two machines were used to conduct the machine learning experiments, namely, a Dell Desktop computer with Intel Core i7-10700 CPU @ 2.90 GHz, Windows 10 enterprise 64-bit OS and 32 GB RAM, and a Dell laptop computer with Intel Core i5-5300U CPU @ 2.30 GHz, Windows 7 enterprise 64-bit OS and 9 GB RAM.

Scikit-learn, a free Python machine learning library, was used to conduct all model training and testing procedures. Other Python libraries, including numpy, pandas, and matplotlib, were also included for data analysis and presentation. Specifically, matplotlib.pyplot was used to generate the box plot of the six features (supplementary scripts/data_boxplot.py). Sklearn.preprocessing.StandardScaler was used to standardize the values of the six features (mean = 0, standard deviation = 1). Sklearn.decomposition.PCA and sklearn.manifold.TSNE were used to perform the PCA (supplementary scripts/pca_training.py) and t-SNE (supplementary scripts/tnse_training.py) analysis, respectively.

To evaluate the performance of all models, the training dataset was first subjected to tenfold cross-validation using multiple machine learning algorithms (Fig. 2). Briefly, sklearn.linear_model.LogisticRegression was used to construct the logistic-regression model (supplementary scripts/lg.py). Sklearn.neighbors.KNeighborsClassifier was used to construct 100 K-NN models with the parameter number of neighbors varying from 1 to 100 (supplementary scripts/knn_training.py). Sklearn.ensemble.RandomForestClassifier was used to construct 100 random forest models with the parameter number of tress varying from 1 to 100 (supplementary scripts/randomforest_training.py).

For MLP, we fixed the number of hidden layers at 2, and scanned all possible combinations of first layer (number of nodes from 1 to 30) and second layer (number of nodes from 1 to 30) using sklearn.neural_network.MLPClassifier (supplementary scripts/mlp_training.py). Additional parameters included: (1) solver = “adam”, (2) alpha = 0.001, (3) random_state = 1, and (4) max_iter = 1000.

For SVM, grid search was implemented for linear kernel (supplementary scripts/svm_training_linear.py) using parameter C = [0.001, 0.01, 0.1, 1, 10], for sigmoid kernel (supplementary scripts/svm_training_sigmoid.py) using C = [0.001, 0.01, 0.1, 1, 10, 100, 1000], and for Gaussian kernel (supplementary scripts/svm_training_gaussian.py) using C = [0.001, 0.01, 0.1, 1, 10, 100, 1000] and gamma = [1000, 100, 10, 1, 0.1, 0.01, 0.001, 0.0001].

The experimental dataset was further subjected to clustering analysis. Specifically, sklearn.cluster.KMeans (supplementary scripts/k_means.py) was used to perform the K-means clustering (k = 2), and sklearn.mixture.GasussianMixture (supplementary scripts/gaussian.py) was used to perform the Gaussian mixture clustering.

Finally, for ensemble models, sklearn.ensemble.VotingClassifier was used to perform the hard and soft voting (supplementary scripts/hard_vote.py, supplementary scripts/soft_vote.py).

### Performance metrics

Performance of different models was evaluated using threshold dependent and independent metrics, which include:

(1) precision: this parameter measures how accurate a model is when predicting cells being at live state.

Precision = TP/(TP + FP), where TP refers to correctly predicted live cells and FP refers to falsely predicted live cells.

(2) recall: this parameter measures the model’s ability to correctly predict live cells from actual live cells.

Recall = TP/(TP + FN), where TP refers to correctly predicted live cells and FN refers to falsely predicted apoptotic cells.

(3) true positive rate (TPR): this parameter measures the model’s ability to correctly predict live cells from actual live cells.

TPR = TP/(TP + FN), where TP refers to correctly predicted live cells and FN refers to falsely predicted apoptotic cells.

(4) false-positive rate (FPR): this parameter measures the model’s level of falsely predicting live cells from actual apoptotic cells.

FPR = FP/(FP + TN), where FP refers to falsely predicted live cells and TN refers to correctly predicted apoptotic cells.

(5) accuracy: this parameter determines the success of correctly predict live and apoptotic cells from overall data.

Accuracy = (TP + TN)/(TP + FP + TN + FN), where TP refers to correctly predicted live cells, FP refers to falsely predicted live cells, FN refers to falsely predicted apoptotic cells, and TN refers to correctly predicted apoptotic cells.

### Reporting summary

Further information on research design is available in the Nature Research Reporting Summary linked to this article.

## Supplementary information

Supplementary Information

Reporting Summary

## Data Availability

All relevant data are available in the online version of the paper (Supplementary_Materials.pdf) and the GitHub repository (https://github.com/yilitexas/MachineLearning). Correspondence and requests for additional materials should be addressed to L.B. (bleris@utdallas.edu).

## Code availability

All relevant scripts are available in the GitHub repository (https://github.com/yilitexas/MachineLearning). Correspondence and requests for additional material should be addressed to L.B. (bleris@utdallas.edu).
