# Supplementary material for: Cell morphology-based machine learning models for human cell state classification
Source: NPJ Syst Biol Appl. 2021 May 26;7:23. doi: 10.1038/s41540-021-00180-y (PMC8155075; doi:10.1038/s41540-021-00180-y)
Supplement: Supplementary file 1 — Supplementary Information [file 41540_2021_180_MOESM1_ESM.pdf]

Supplementary Materials

Supplementary Figure 1

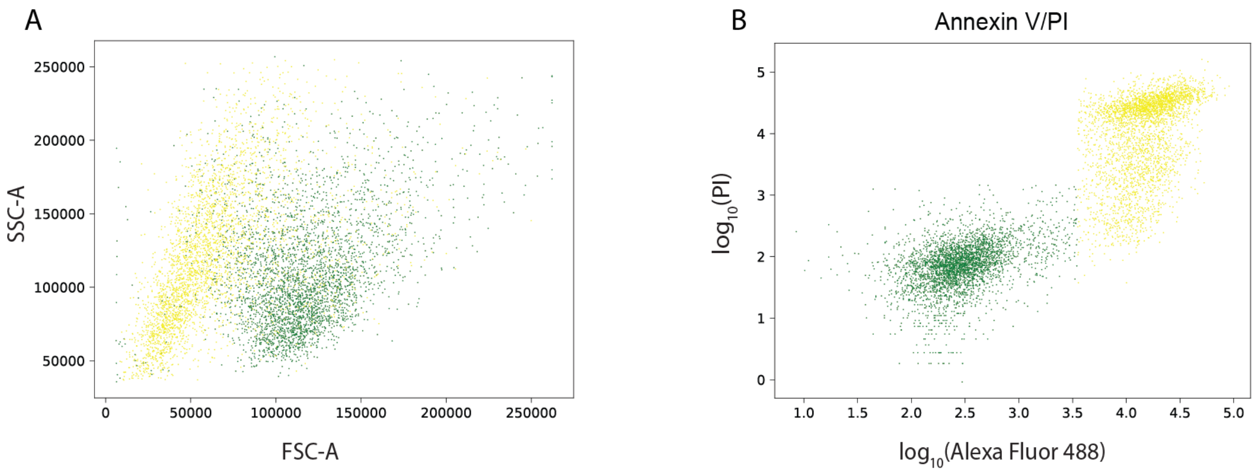

Supplementary Figure 1. Analysis of the training dataset. (A) SSC-A vs. FSC-A plot, (B) Labeling of live and apoptotic cell subpopulations using the Annexin V/PI apoptosis assay (green: live cells, yellow: apoptotic cells).

Supplementary Figure 2

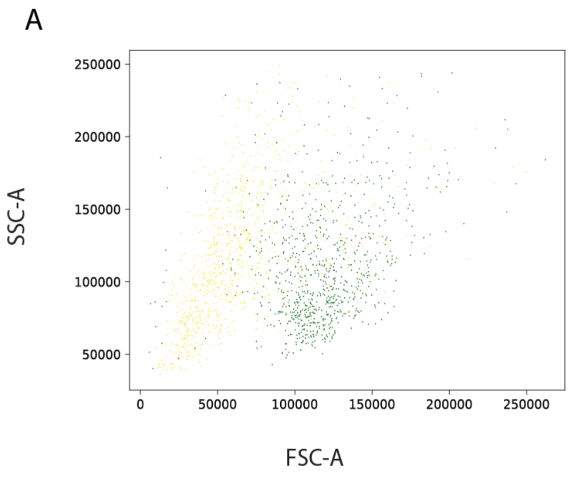

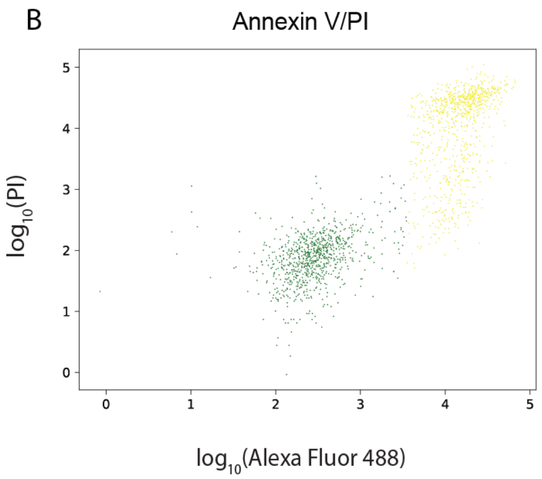

Supplementary Figure 2. Analysis of the testing dataset. (A) SSC-A vs. FSC-A plot, (B) Labeling of live and apoptotic cell subpopulations using the Annexin V/PI apoptosis assay (green: live cells, yellow: apoptotic cells).

Supplementary Figure 3

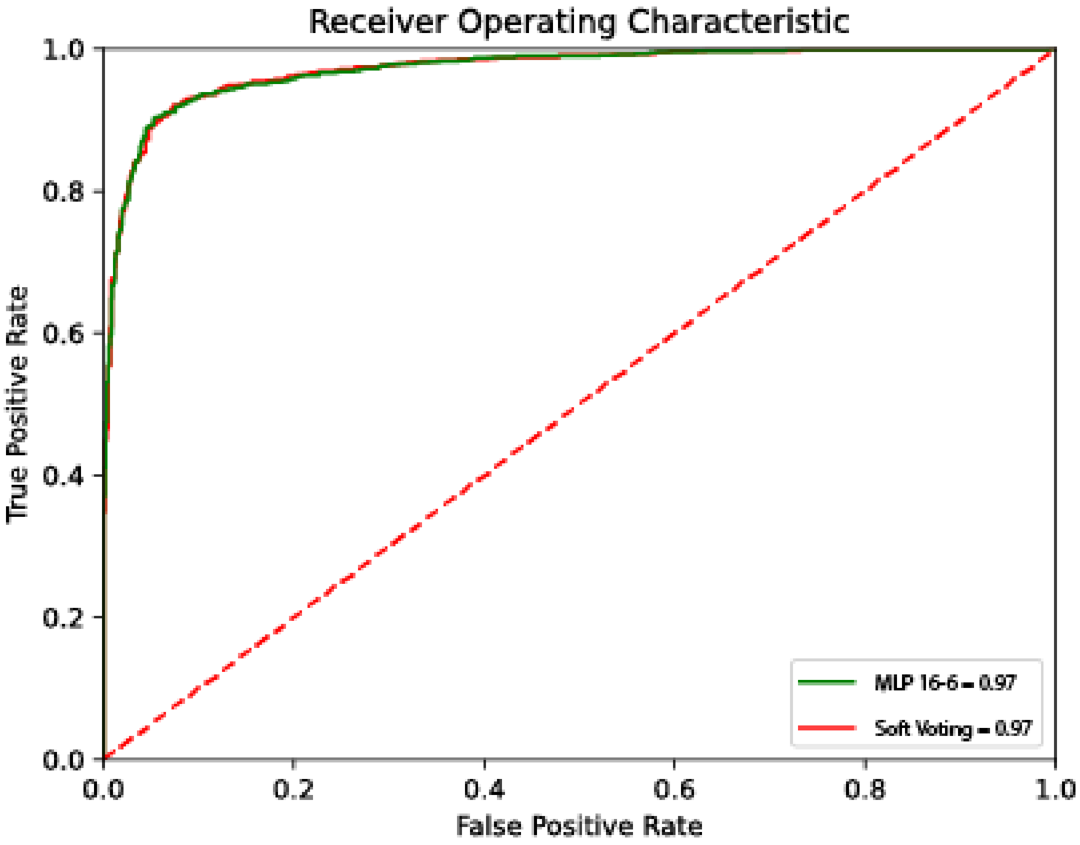

Supplementary Figure 3. Predictive performances of the ensemble models. The ensemble models based on the 3 individual candidate models using hard voting or soft voting did not significantly improve the predictive performance compared to the MLP 16-6 model.

Supplementary Figure 4

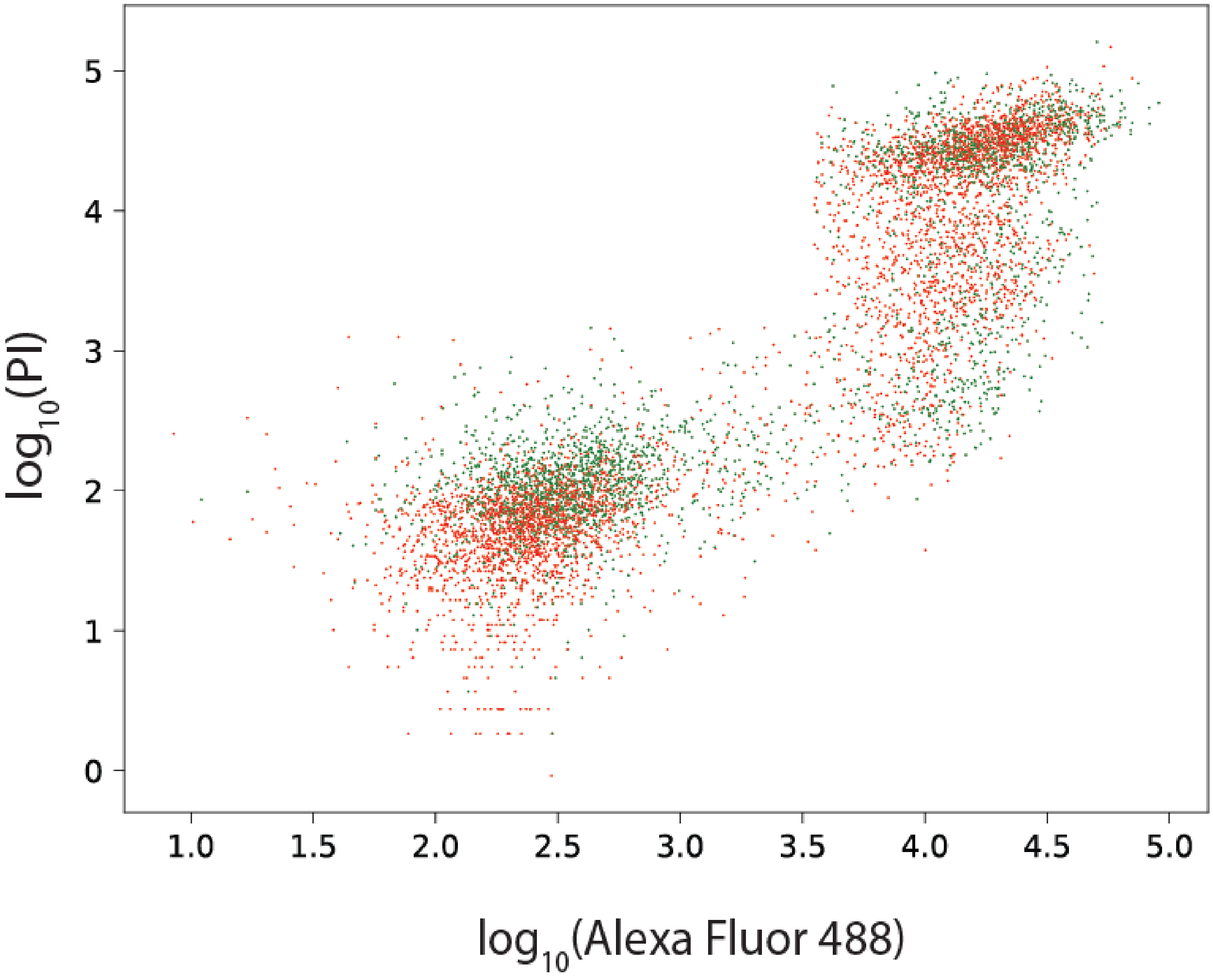

Supplementary Figure 4. Clustering using k-means algorithm on non-standardized training dataset.

Supplementary Figure 5

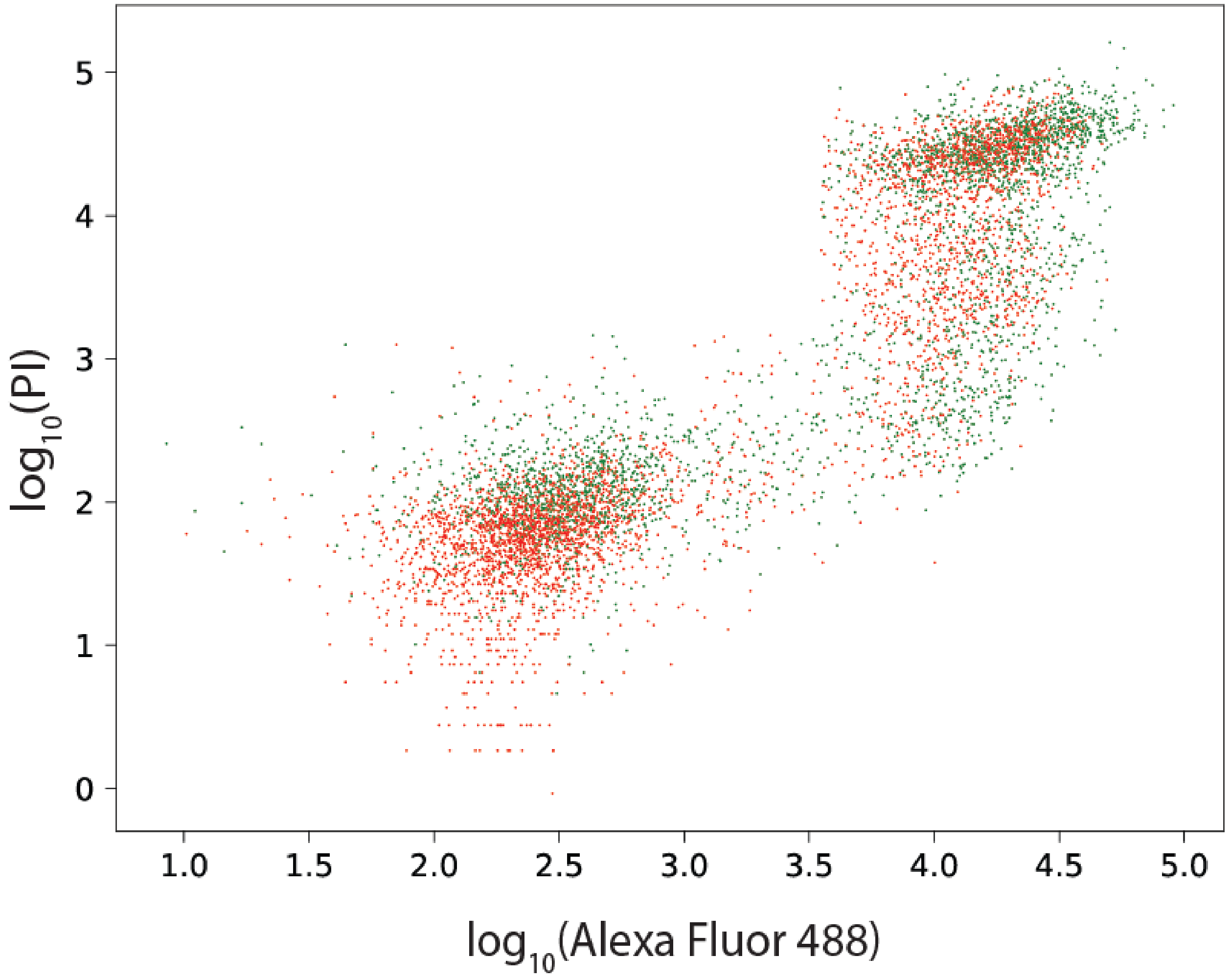

Supplementary Figure 5. Clustering using Gaussian mixture algorithm on non-standardized training dataset.

Supplementary Figure 6

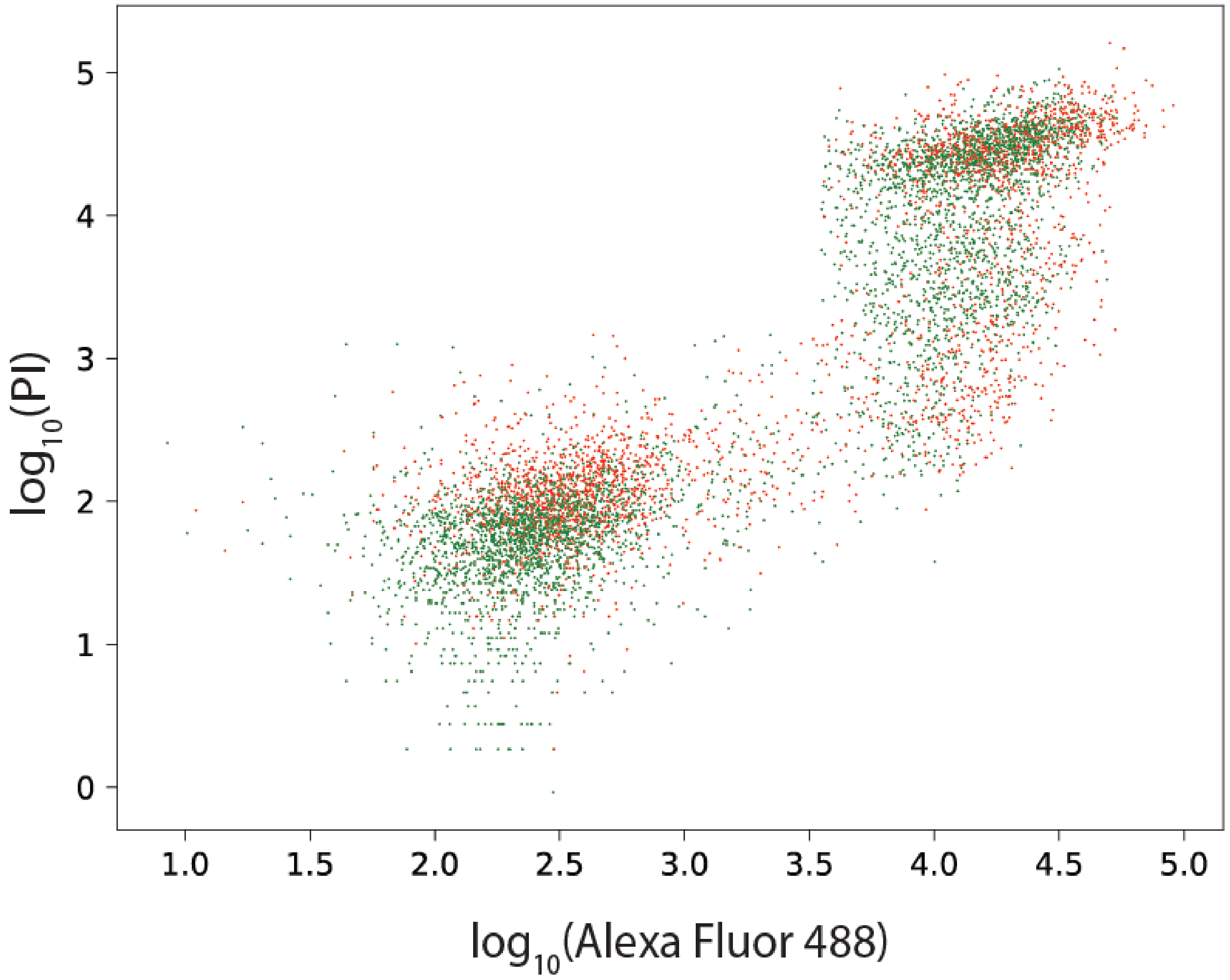

Supplementary Figure 6. Clustering using k-means algorithm on standardized training dataset.

Supplementary Figure 7

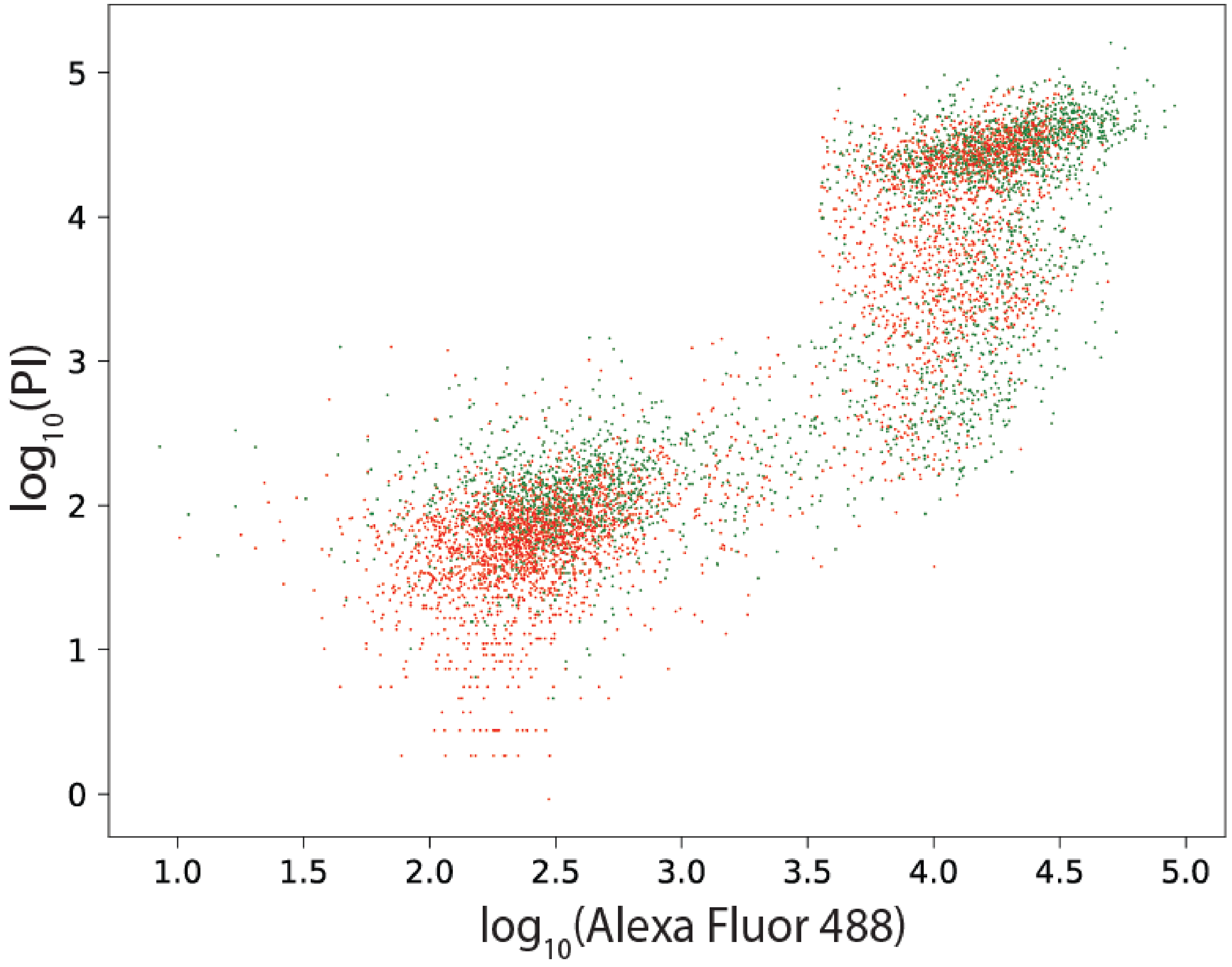

Supplementary Figure 7. Clustering using Gaussian mixture algorithm on standardized training dataset.

Supplementary Figure 8

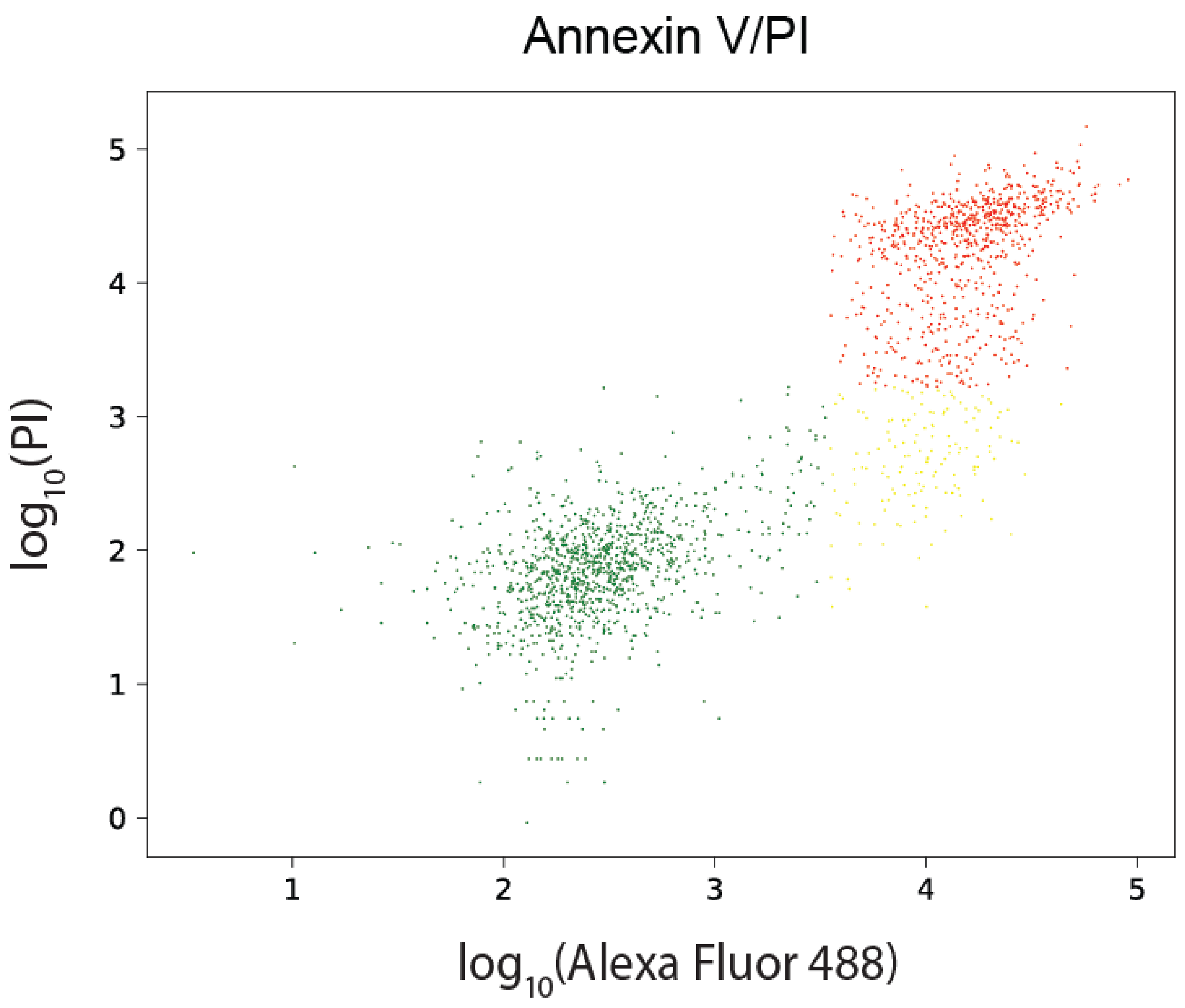

Supplementary Figure 8. Labeling of live, early-apoptotic and late-apoptotic cell subpopulations using the Annexin V/PI apoptosis assay (green: live cells, yellow: early-apoptotic cells, red: late-apoptotic cells).

Supplementary Figure 9

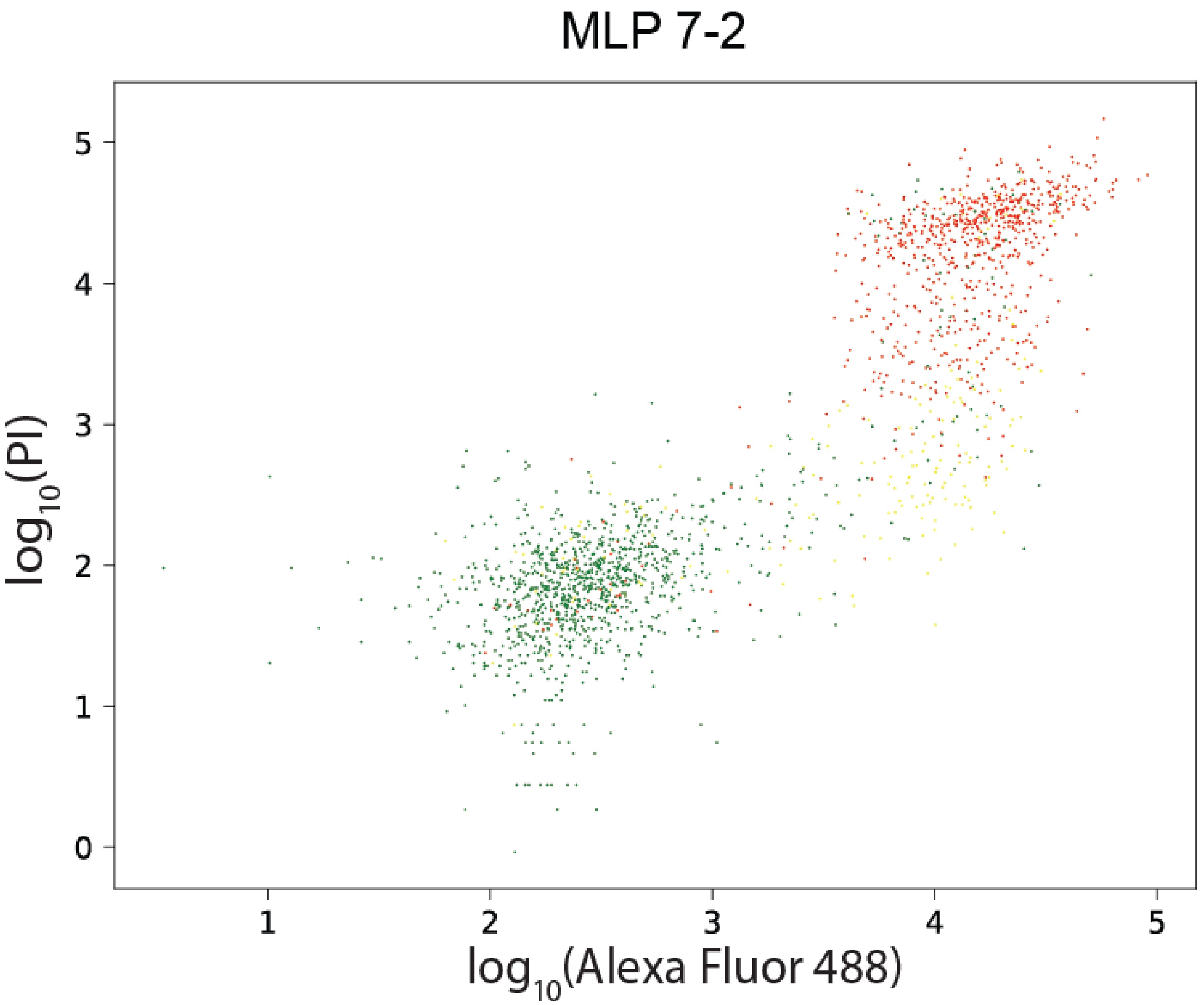

Supplementary Figure 9. Prediction of live, early-apoptotic and late-apoptotic cell subpopulations using a MLP-based learning model (MLP 7-2, number of nodes in the 1st layer = 7, number of nodes in the 2nd layer = 2, green: live cells, yellow: early-apoptotic cells, red: late-apoptotic cells).

Supplementary Figure 10

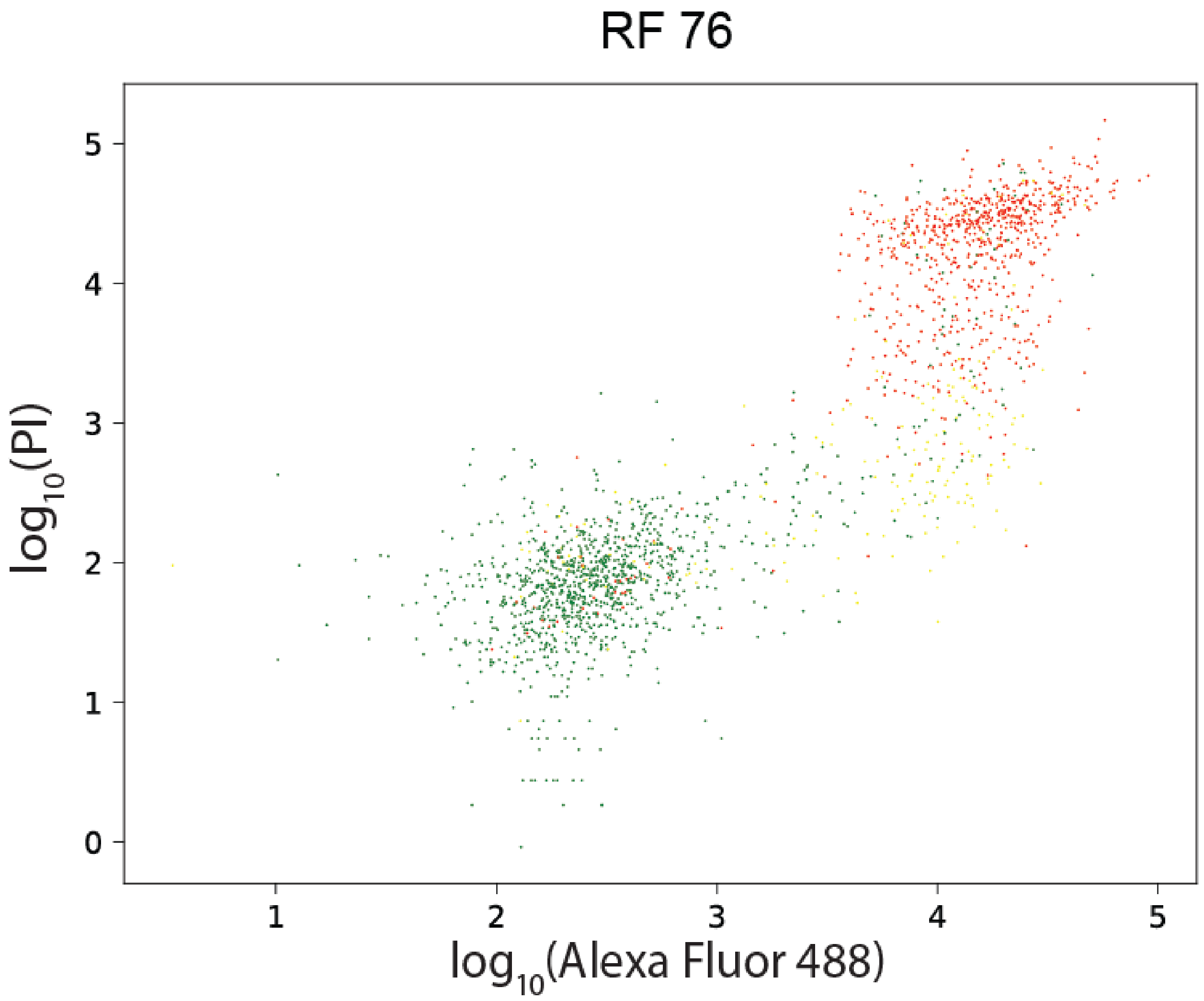

Supplementary Figure 10. Prediction of live, early-apoptotic and late-apoptotic cell subpopulations using a random forest-based learning model (RF 76, number of trees = 76, green: live cells, yellow: early-apoptotic cells, red: late-apoptotic cells).

Supplementary Scripts. The MLP 16-6 model and other main python scripts used in building and evaluating the predictive models in this study.

https://github.com/yilitexas/MachineLearning/tree/main/Supplementary_Scripts

Supplementary Table 1. The training dataset. The training dataset contained 6,828 cells, of which 3,411 were live and 3,417 were apoptotic.
https://github.com/yilitexas/MachineLearning/blob/main/supplementary_table1.xls

Supplementary Table 2. The testing dataset. The testing dataset contained 1,708 cells, of which 857 were live and 857 were apoptotic. https://github.com/yilitexas/MachineLearning/blob/main/supplementary_table2.xls

feature	FSC-A	FSC-H	FSC-W	SSC-A	SSC-H	SSC-W
mean	91461.29759	64077.81678	92856.09651	116136.1841	89860.60984	83829.85344
standard deviation	42073.01065	27018.88786	9980.780925	42723.22343	29737.5374	6146.286279

Supplementary Table 3. The mean and standard deviation values of the 6 features in the training dataset.

Supplementary Table 4. The mean and standard deviation values of 10-fold cross validation accuracy using the random forest algorithm.

https://github.com/yilitexas/MachineLearning/blob/main/supplementary_table4.xls

Supplementary Table 5. The mean and standard deviation values of 10-fold cross validation accuracy using the k-NN algorithm.

https://github.com/yilitexas/MachineLearning/blob/main/supplementary_table5.xls

Supplementary Table 6. The mean and standard deviation values of 10-fold cross validation accuracy using the MLP algorithm.

https://github.com/yilitexas/MachineLearning/blob/main/supplementary_table6.xls

Supplementary Table 7. The mean and standard deviation values of 10-fold cross validation accuracy using the SVM algorithm.

https://github.com/yilitexas/MachineLearning/blob/main/supplementary_table7.xls

Supplementary Table 8. The predictive performances of the 96 candidate models which passed both filtering conditions. https://github.com/yilitexas/MachineLearning/blob/main/supplementary_table8.xls

Supplementary Table 9. The training dataset with 3 labels. The training dataset contained 7,992 cells, of which 4,607 were live, 552 were early-apoptotic, and 2,833 were late-apoptotic.
https://github.com/yilitexas/MachineLearning/blob/main/supplementary_table9.xls

Supplementary Table 10. The testing dataset with 3 labels. The testing dataset contained 1,998 cells, of which 1,115 were live, 147 were early-apoptotic, and 736 were late-apoptotic. https://github.com/yilitexas/MachineLearning/blob/main/supplementary_table10.xls

Supplementary Table 11. The training dataset containing synthetic samples for early- and late-apoptotic cells. The synthetic samples for minority classes (early- and late-apoptotic cells) were generated using the SMOTE algorithm. The training dataset contained 13,821 cells, of which 4,607 were live, 4,607 were early-apoptotic, and 4,607 were late-apoptotic.
https://github.com/yilitexas/MachineLearning/blob/main/supplementary_table11.xls

Supplementary Table 12. The predictive performances of the 5 candidate MLP models which passed the filtering condition. https://github.com/yilitexas/MachineLearning/blob/main/supplementary_table12.xls

Supplementary Table 13. The predictive performances of the 49 candidate random forest models which passed the filtering condition.
https://github.com/yilitexas/MachineLearning/blob/main/supplementary_table13.xls

Supplementary Table 14. Comparison of our study and other protocols predicting cell types and states.

https://github.com/yilitexas/MachineLearning/blob/main/supplementary_table14.xls
